# Involvement of IL-1 in the Maintenance of Masseter Muscle Activity and Glucose Homeostasis

**DOI:** 10.1371/journal.pone.0143635

**Published:** 2015-11-24

**Authors:** Ko Chiba, Masahiro Tsuchiya, Masashi Koide, Yoshihiro Hagiwara, Keiichi Sasaki, Yoshinori Hattori, Makoto Watanabe, Shunji Sugawara, Makoto Kanzaki, Yasuo Endo

**Affiliations:** 1 Division of Aging and Geriatric Dentistry, Tohoku University Graduate School of Dentistry, Sendai, Japan; 2 Division of Oral Molecular Regulation, Tohoku University Graduate School of Dentistry, Sendai, Japan; 3 Kansei Fukushi Research Institute, Tohoku Fukushi University, Sendai, Japan; 4 Division of Oral Diagnosis, Tohoku University Graduate School of Dentistry, Sendai, Japan; 5 Department of Orthopaedic Surgery, Tohoku University Graduate School of Medicine, Sendai, Japan; 6 Division of Advanced Prosthetic Dentistry, Tohoku University Graduate School of Dentistry, Sendai, Japan; 7 Tohoku University Graduate School of Biomedical Engineering, Sendai, Japan; Ohio State University Medical Center, UNITED STATES

## Abstract

Physical exercise reportedly stimulates IL-1 production within working skeletal muscles, but its physiological significance remains unknown due to the existence of two distinct IL-1 isoforms, IL-1α and IL-1β. The regulatory complexities of these two isoforms, in terms of which cells in muscles produce them and their distinct/redundant biological actions, have yet to be elucidated. Taking advantage of our masticatory behavior (Restrained/Gnawing) model, we herein show that IL-1α/1β-double-knockout (IL-1-KO) mice exhibit compromised masseter muscle (MM) activity which is at least partially attributable to abnormalities of glucose handling (rapid glycogen depletion along with impaired glucose uptake) and dysfunction of IL-6 upregulation in working MMs. In wild-type mice, masticatory behavior clearly increased IL-1β mRNA expression but no incremental protein abundance was detectable in whole MM homogenates, whereas immunohistochemical staining analysis revealed that both IL-1α- and IL-1β-immunopositive cells were recruited around blood vessels in the perimysium of MMs after masticatory behavior. In addition to the aforementioned phenotype of IL-1-KO mice, we found the IL-6 mRNA and protein levels in MMs after masticatory behavior to be significantly lower in IL-1-KO than in WT. Thus, our findings confirm that the locally-increased IL-1 elicited by masticatory behavior, although present small in amounts, contributes to supporting MM activity by maintaining normal glucose homeostasis in these muscles. Our data also underscore the importance of IL-1-mediated local interplay between autocrine myokines including IL-6 and paracrine cytokines in active skeletal muscles. This interplay is directly involved in MM performance and fatigability, perhaps mediated through maintaining muscular glucose homeostasis.

## Introduction

Prolonged physical exercise results in fatigue, stiffness, and pain in working skeletal muscles. The masseter muscles (MMs), one of the major multipennate muscles, differ in structure and function from other skeletal muscles [1]. Dysfunction of masticatory MMs is seen in temporomandibular disorders (TMD), and is believed to result from abnormal activity, including bruxism and/or prolonged clenching [2].The increase of physical exhaustion for the energetic consumption like an exercise causes the acute fatigue, whereas the impairment to manage a certain level of force is the cause of chronic fatigue [3,4]. Moreover, muscle fatigue can be due to impaired excitation-contraction coupling, and dysregulation of energy homeostasis resulting in greater metabolic costs in spite of adequate nutrient supply to the working muscles [4–6].

While susceptibility to fatigue is common among the elderly, it is also an early sign for muscle disorders such as neuromuscular disease, chronic fatigue syndrome, and TMD [7–10]. Multiple studies of self-reported fatigue across the lifespan found that fatigability progressively increased with age, but was also a crucial issue among younger adults. For instance, reduced muscle power has been reported in 13% of young men (20–30 years of age), 19% of old men (in their 60s), and 24% of very old men (> 70 years) [11,12]. Muscle fatigability is strongly linked to interferences with daily activities and reduced quality of life [13,14], and fatigue of the MMs specifically leads to decreased chewing ability, which can directly impact dietary habits and lifestyle [15].

Physical exercise has been shown to increase the plasma levels of various proinflammatory cytokines such as IL-1β, IL-6, and TNF-α [16–19]. Specifically, IL-6, which is considered to be a typical “myokine” as it is a cytokine produced and secreted by muscle cells, has been suggested to play an important role as an “energy sensor” in exercise-related metabolic changes [16–18]. We have previously reported that MM activity led to the production and release of IL-6 in mice, and our data further suggested that the MM-derived IL-6 helped MM muscle fatigue recovery by maintaining glucose homeostasis [20].

Although the role of IL-1β in supporting skeletal muscle activity during exercise was originally reported over thirty years ago (8), its role has remained controversial because there are little exercise-dependent increases in IL-1β plasma levels [16–18]. Nevertheless, previous studies have provided evidence that eccentric bicycle exercise significantly increased IL-1 activity in the plasma of human subjects [21,22]. Interestingly, a historic report by Cannon and Kluger demonstrated that mononuclear leukocytes obtained from human subjects after exercise released pyrogenic factors into culture medium, which were presumably IL-1 proteins [23]. Both isoforms of IL-1, IL-1α and IL-1β, function as proinflammatory cytokines, and their production is controlled by a complex regulatory mechanism. However, several lines of evidence have also suggested that both IL-1 isoforms exhibit a variety of metabolic effects [24,25]. Recent studies have shown that IL-1α is constitutively expressed in some cell types and is released upon cell damage, by which it can then stimulate other cell types in a paracrine manner to produce IL-1β [26,27].

Thus, it is not yet clear (a) whether exercise does indeed stimulate skeletal muscle fibers or related cell-types to produce IL-1, and (b) whether IL-1 is involved in supporting muscle activity. In the current study, a unique model that we established for quantitatively analyzing masticatory muscle fatigue in mice was utilized to address these questions [28,29], in which we quantified MM activity in mice deficient in both IL-1α and IL-1β (IL-1-KO mice), and compared this to the MM activity in wild-type (WT) control mice. It should be noted that there were no physical or health abnormalities apparent in the IL-1-KO mice [30], though their muscle fatigability had not been determined.

## Materials and Methods

### Animal Studies

In the design and performance of these experiments, the principles of laboratory animal care (NIH Publication No. 86–23, revised 1985) were followed, and specific national laws were obeyed. All aspects of handling, care, and use of animals were approved by the Ethics Committee for Animal Experiments, Tohoku University (2011DNA-36, 2012DNA-11, and 2013DNA-041). WT control BALB/c mice were obtained from CLEA-Japan (Tokyo, Japan). Homozygous BALB/c IL-1-KO mice (deficient in both IL-1α and IL-1β) were established from original IL-1α-KO and IL-1β-KO mice [31] by backcrossing to BALB/c mice, and maintained in our animal facilities. Deficiency in IL-1α/β expression was routinely confirmed by RT-PCR analysis (S1 Fig). The mice used for the present experiments were 7–8 week old males. All mice were allowed standard food pellets (LabMR Stock; Nihon Nosan Inc, Yokohama, Japan) and tap water *ad libitum* in an air-conditioned room at 23 ± 1°C with 55 ± 5% relative humidity and a 12 h light-dark cycle (lights on at 07:00 a.m.). Recombinant mouse IL-1α and β (rm IL-1α and rm IL-1β) was purchased from BioLegend (San Diego, CA, USA). rm IL-1β was intravenously injected into WT mice (n = 5–6) at a dose of 5 ng per g body weight (BW). Then, 1 h later the mice were euthanized by decapitation under anesthesia via inhalatory isoflurane, in accord with the national Standards Relating to the Care and Management of Laboratory Animals and Relief of Pain (Notification No.88 of the Ministry of the Environment, Japan, April 28, 2006).

### Evaluation of MM Activity

**Fig 1:**
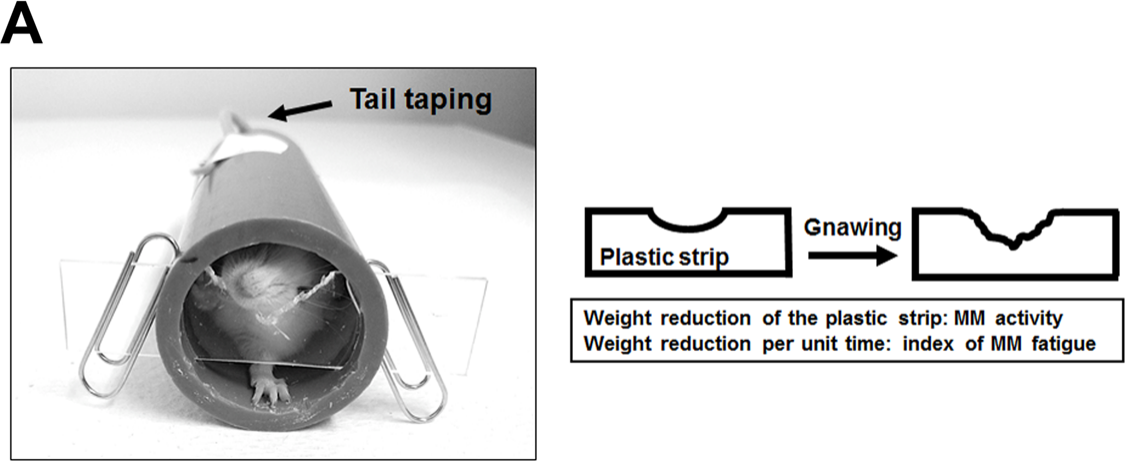
(A) The R+G+ model for evaluation of MM activity. A mouse is restrained (R+) within a cylinder (internal diameter 2.5 cm), of which the front end is blocked with a thin plastic strip (width 1.5 cm, thickness 1.0 mm, length 10 cm). A mouse that can reach the strip gnaws on it (G+) to escape. Taping the tail of a mouse to the cylinder denies the mouse access to the strip. We termed the former condition R+G+ and the latter R+G-, while R-G- means that mice are put in a cage without restraint. The reduction in the weight the strip reflects the MM activity for the 1 h period the mouse is in place, and the decline in that parameter over time is used to calculate a fatigue index for MMs.

When a mouse is restrained (a condition termed “R+”) within a cylinder, the mouse gnaws (termed “G+”) at a plastic strip blocking one end in an attempt to escape (a condition called “R+G+”) (Fig 1A) as previously described [28,29]. It is difficult to quantify the physiological fatigue of this voluntary masticatory behavior because it involves complex isometric-isotonic muscular contractions [1,32], although the weight-reduction in the strip can be used as an index of MM activity. In the present study, the weight-reduction in the strip resulting during the hour a mouse was restrained was representative of the MM activity for that period, and a decline in that parameter during prolonged R+G+ provided an index of fatigue as previously described [28]. The following two groups were used as controls: “R+G-,” in which the tail was fastened by tape to the cylinder at such a position that the mouse was unable to reach the strip; and “R−G−,” in which the mice were kept in their home cages without food or water for the duration of the experimental period. Each group contained 12 mice, and all experiments were performed in the evening.

### Blood Glucose

The tail veins were pierced, and the extruded blood (about 5 μl) was directly applied to a glucometer (Accu-Chek Advantage, Roche Diagnostics K.K., Tokyo, Japan). Each group contained 4–5 mice.

### Glycogen in MMs

Tissue glycogen was quantified as described previously [33]. Briefly, glycogen was separated from MMs (from 12–16 mice) and hydrolyzed to glucose (using H_2_SO_4_ at 100°C for 2 h). After neutralization of the reaction mixture with NaOH, glucose was measured using the Wako Glucose-CII test (Wako Pure Chemical Industries, Osaka, Japan).

### Glucose Uptake by MMs

Since 2-deoxy-D-glucose (2DG) is taken up via glucose transporters, but not metabolized by the glycolytic pathway, 2DG has been used as a marker for evaluating the amount of glucose uptake. Here, ^14^C-2DG (5 μCi/ml) was intraperitoneally injected (0.1 ml per 10 g BW, n = 10–12), and 30 min later, MMs were removed and subjected to analysis of ^14^C-2DG content using the method reported by Ferré et al. [34].

### Quantitative PCR (qPCR) Analysis

Total RNA was extracted from MMs (from 6–8 mice in each group) with TRIzol reagent (Invitrogen, CA, USA). cDNA was reverse transcribed using a Transcriptor First Strand cDNA Synthesis Kit (Roche Diagnostics, Minneapolis, MN, USA) with oligo-dT primers. The cDNA was subjected to qPCR amplification in a BioRad CFX96 qPCR system (Bio-Rad, Hercules, CA, USA). The relative expression of target genes was determined by the 2^–ΔCT^ method [35]. Primers and their sequences were as follows: IL-1α: forward 5′-GAC CGA CCT TTT CTT CTG-3′ and reverse 5′-AGG TGC ACC CGA CTT TGT TCT T-3′; IL-1β: forward 5′-GCA CCT TCT TTT CCT TCA TCT TTG-3′ and reverse 5′-GTT GTT CAT CTC GGA GCC TGT-3′; IL-6: forward 5′-AAC CAC GGC CTT CCC TAC TT-3′ and reverse 5′-CCA TTG CAC AAC TCT TTT CTC ATT-3′; EF1α1 (internal reference control): forward 5′-ATT CCG GCA AGT CCA CCA CAA-3′ and reverse 5′-CAT CTC AGC AGC CTC CTT CTC AAA C-3′.

### Measurement of IL-1 and IL-6

Tissue extracts were prepared as described previously [36], and were subjected to cytokine measurement. Each group contained 10–15 mice. ELISA kits were used for measuring IL-1α (R&D Systems, Inc, Minneapolis, MN, USA), IL-1β (Thermo Fisher Scientific Inc, Rockford, IL, USA), and IL-6 (Thermo).

### Immunohistochemical (IHC) Analysis for IL-1α and β

After 2 h of masticatory behavior, MMs (dissected from 2–3 mice in each group) were resected and fixed in 4% paraformaldehyde overnight at 4°C. Samples were then dehydrated and embedded in paraffin. Serial sections were used for immunostaining as previously described [37]. After deparaffinization, antigen retrieval was performed with proteinase K solution for 5 minutes at 37°C, followed by incubation with the primary antibody for IL-1α (1: 40 dilution; Abcam, Cambridge, MA, USA, #ab7632) and for IL-1β (1: 500 dilution; Abcam, #ab9722) at 4°C overnight. Next, the sections were incubated with a secondary biotinylated goat anti-rabbit antibody (1: 1,000 dilution; Vector Laboratories, Tokyo, Japan), and then visualized using the Vectastain ABC kit (Vector Laboratories). Fluorescent double-labeled IHC was carried out using the primary antibody for granulocyte-differentiation antigen (Gr)-1 (1:500 dilution; BioLegend, San Diego, CA, USA, clone RB6-8C5), a major marker for granulocytes and partially for subsets of endothelial cells [38,39], and DAPI counterstaining. The secondary antibodies used were Alexa^488^-conjugated goat-anti rabbit and Alexa^555^-conjugated goat anti-rat (1:750 dilution; Molecular Probes, Eugene, OR, USA). The slides were examined with a Leica TCS SP8 confocal laser scanning microscope (Leica Microsystems, Mannheim, Germany). The specificity of immune reactions was verified by replacing the primary antibody with rabbit IgG (Abcam, #ab27472) as an isotype control [40].

### Cell Culture

To examine the possible involvement of IL-1 in IL-6 production, the C2C12 mouse myoblast cell line was maintained in DMEM supplemented with 10% FBS, 100 μg/ml penicillin, and 100 μg/ml streptomycin (growth medium) at 37°C with 5% CO_2_. For biochemical studies, the cells were grown on 8-well plates (BD Biosciences, San Jose, CA, USA) at a density of 2.5 × 10^4^ cells/well in 2.5 ml of growth medium. Three days after plating, the cells had reached approximately 80–90% confluency (day 0). Differentiation into myotubes was then induced by replacing the growth medium with DMEM (4.5 g/liter glucose) supplemented with 2% FBS, 1 nM insulin, 30 μg/ml penicillin, and 100 μg/ml streptomycin (differentiation medium) [41]. The differentiation medium was changed every 24 h, and the differentiated cells (at days 4 and 5) were used for experiments. The cells were then incubated for 2 h in DMEM with or without 20–2000 pg/ml rm IL-1α or β, and then the culture medium was collected and IL-6 levels were measured using an ELISA kit (see above).

### Data Analysis

All statistical analyses were performed using InStat software. Experimental values are given as the mean ± standard error (SE). The statistical significance was assessed either by a Student’s unpaired *t*-test (for comparing two means) or by a Bonferroni multiple-comparison test after two-way ANOVA (for comparing three or more means). *P*-values less than 0.05 were considered significant.

## Results

### Effects of MM Activity on IL-1α and β Production

**Fig 2:**
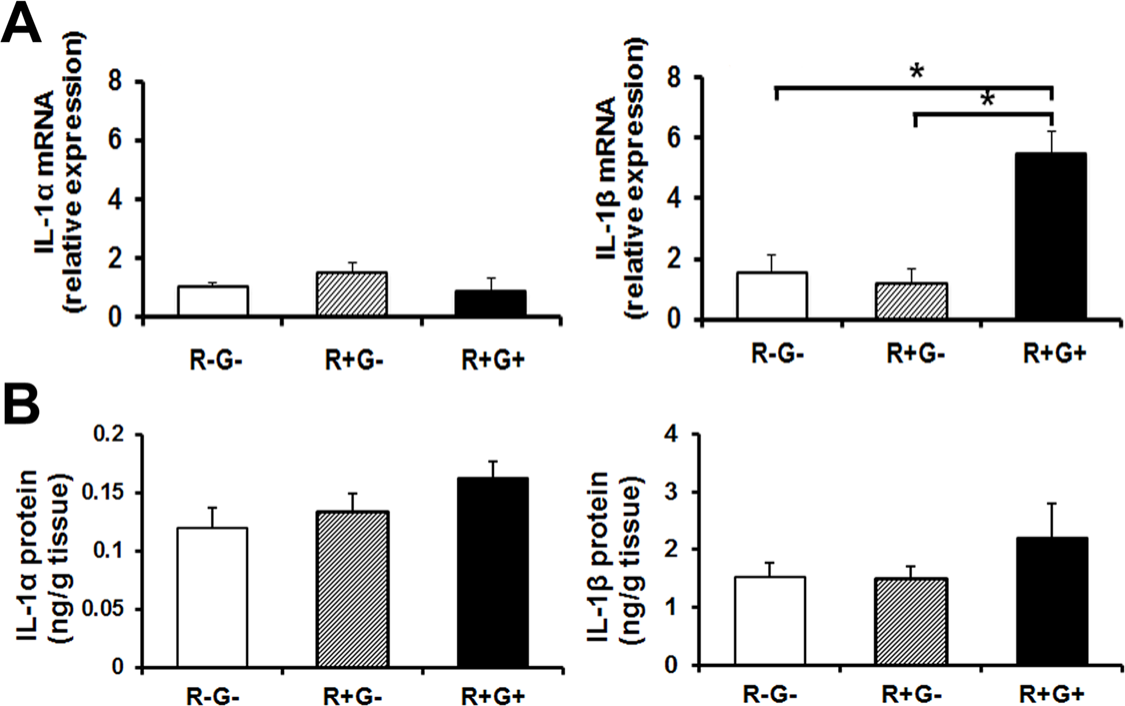
IL-1 mRNA and protein levels in response to R+G+, and distribution in MMs. (A) R+G+ increased mRNA expression of IL-1β (but not of IL-1α) in WT-MMs. WT mice were subjected to 1 h of R+G- or R+G+, and IL-1α and IL-1β mRNA expression in MMs was analyzed via qPCR (n = 6–8). Experimental values are given as the mean ± SE. *P < 0.05. (B) R+G+ increased the level of IL-1α and β protein in MMs. WT mice were subjected to 1 h of R-G-, R+G- or of R+G+, and the IL-1α and β proteins in their MMs were analyzed via ELISA. Experimental values are given as the mean ± SE (n = 10–15).

First, we examined whether masticatory behavior induced IL-1 expression in MMs. As shown in Fig 2A, 1 h of R+G+ behavior significantly induced mRNA expression of IL-1β (Fig 2A *right*), but not IL-1α (Fig 2A *left*) in MMs, whereas no induction was observed in the MMs from R-G- or R+G- mice. Despite the increase in IL-1β mRNA expression in the R+G+ group, we could not detect significant increases in IL-1β protein in homogenates of MMs via ELISA, although there was an increase, albeit not significant, after 1 h of masticatory behavior (Fig 2B). Similar results were observed for mRNA expression and protein levels of IL-1α/β in MMs after 2 h of R+G+ behavior (data not shown).

### R+G+ Behavior Increased IL-1-positive Cells in MMs

**Fig 3:**
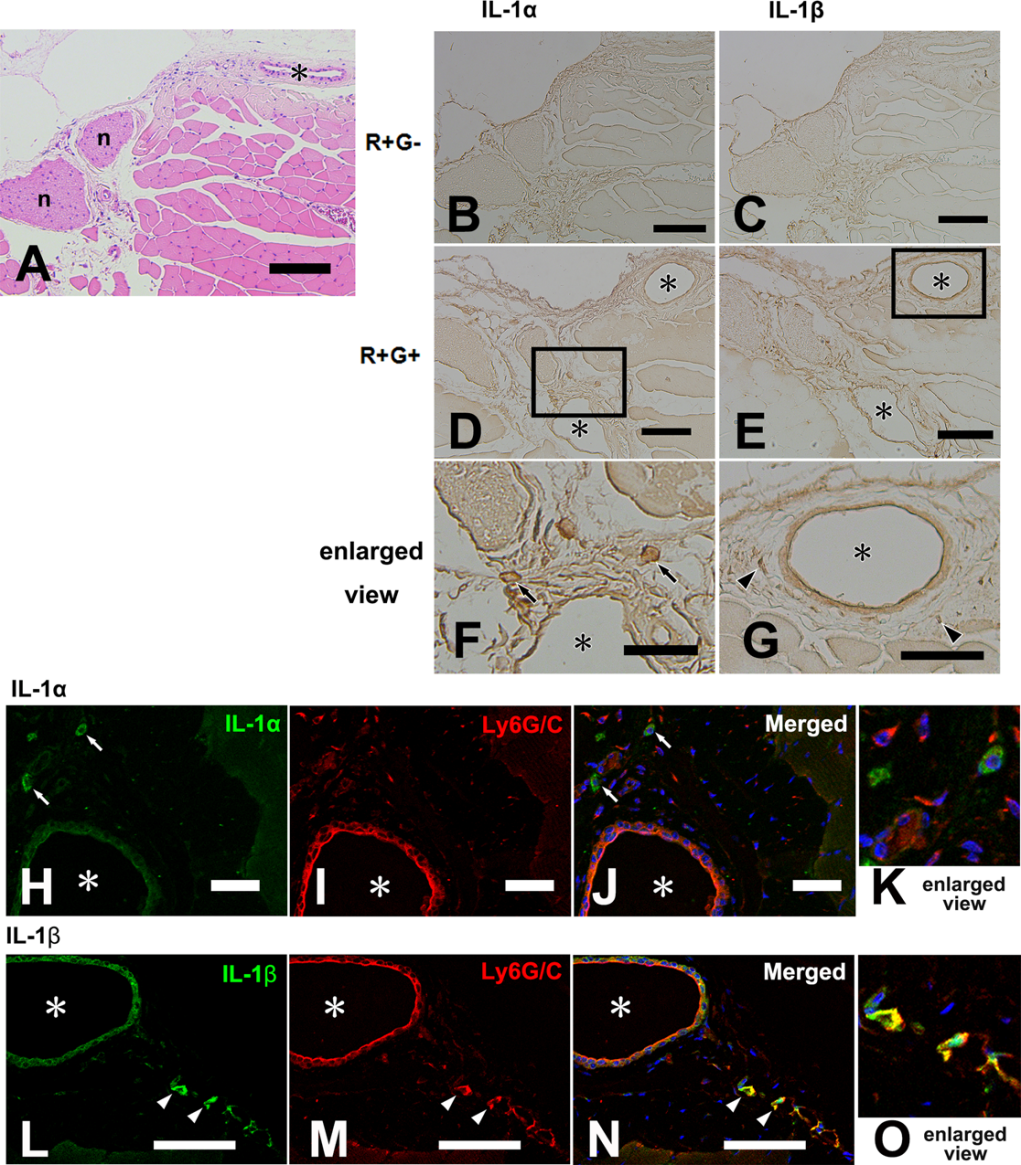
IL-1 immunolocalization in MMs with or without 2 h of R+G+ activity. (A) Hematoxylin-eosin staining for MM (R+G-); (B and C) immunostaining of IL-1α and β for MMs of mice in the R+G- group; (D and E) immunostaining of IL-1α and β for MMs of the mice in the R+G+ group; (F and G) enlarged views of rectangles indicated in (D) and (E), respectively; (H to O) confocal image showing immunoreactivity for IL-1 protein (green, H to K: IL-1α and L to O: IL-1β) and Ly6G/C (red) separately (H, I, L, and M) and as a merged image (J and N), respectively. Examples of immunopositive cells are indicated by arrows (IL-1α in F, H, and J) or by arrowheads (IL-1β in G, L, and N). n: nerve and asterisk: blood vessel; Bars = 100 μm for (A to E), and 50 μm for (F to N).

While both IL-1α and β protein levels in whole MMs increased after R+G+ behavior, they did not reach statistical significance (Fig 2B). Interestingly however, IHC analysis revealed that staining of IL-1α and β in MMs after 2 h of masticatory behavior led to distinct distribution patterns between groups (Fig 3). Histological view of the MM with hematoxylin and eosin staining (H-E) shows typical skeletal muscle with perimysium including nerve and blood vessels (Fig 3A). Although staining of IL-1α and IL-1β was similar within the skeletal muscle fibers of the R+G- (Fig 3B and 3C) and R+G+ (Fig 3D and 3E) groups, IL-1α- and β-positive cells were localized in the perimysium of the MMs only in the R+G+ group, especially around blood vessels (Fig 3F and 3G). To further examine the cellular phenotype of IL-1-positive cells, we performed fluorescent double-labeled IHC with respect to the colocalization of Gr-1, a major marker for migrating neutrophils. Gr-1 signals were clearly observed in IL-1β-positive cells and endothelial cells (Fig 3L to 3O), but not in IL-1α-positive cells (Fig 3H to 3K). Additionally, cells in the MMs of the R+G- group did not share these characteristics. These results indicate that (i) in spite of low concentrations, IL-1 may play a focal role in skeletal muscle activities, and (ii) IL-1 may be produced within MMs after masticatory behavior, potentially by connective tissue cells and/or immune cells recruited from the circulation.

### IL-1-KO-MMs are Easily Fatigued by Masticatory Activity

**Fig 4:**
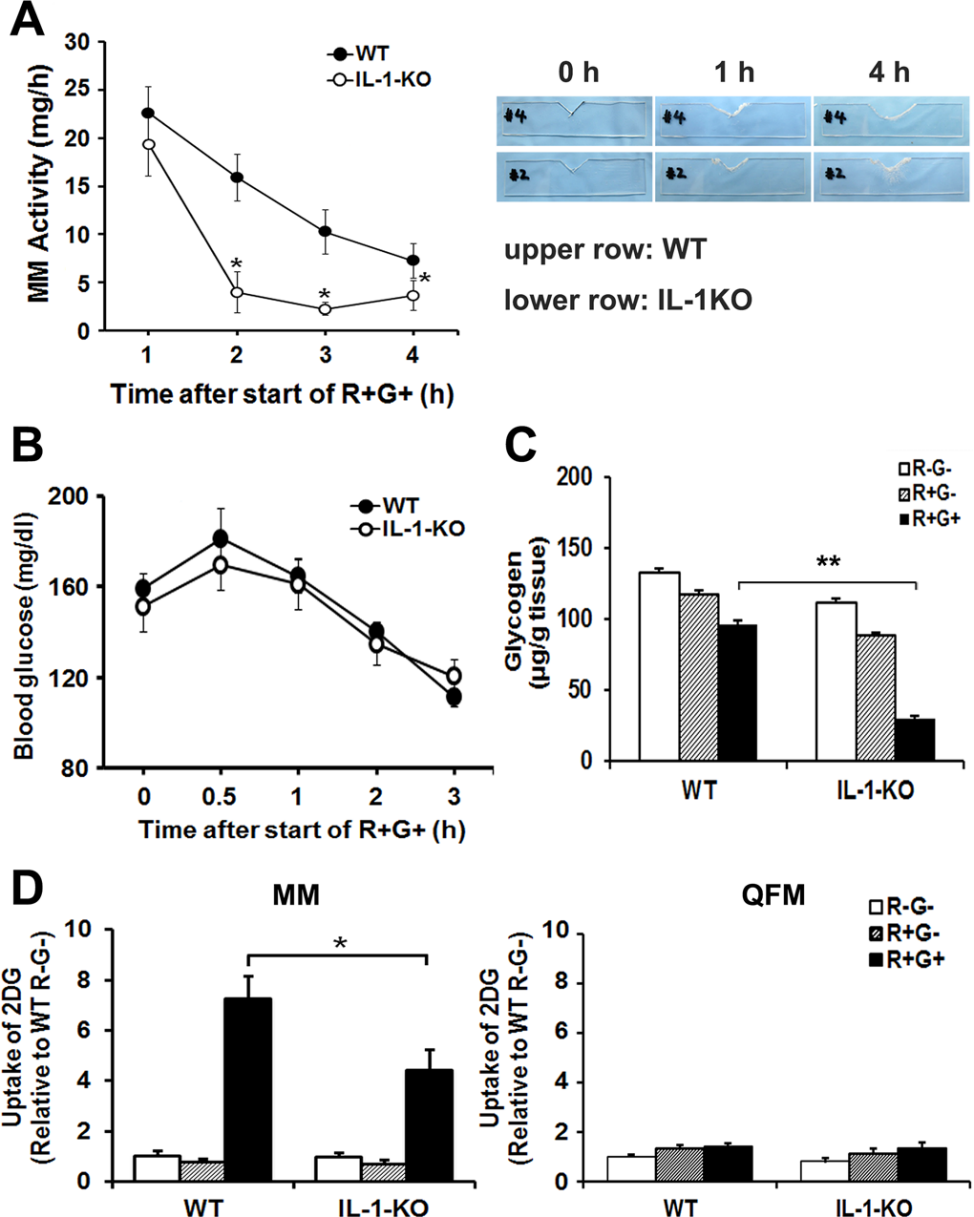
MM activity and glucose metabolism in IL-1-KO mice during R+G+. (A) IL-1-KO-MMs were easily fatigued in the R+G+ condition. WT and IL-1-KO mice (n = 12) were subjected to R+G+, and the MM activity was measured. Typical examples of plastic plates gnawed on during R+G+ by WT or IL-1-KO mice are shown on the right. Experimental values are given as the mean ± SE. *P < 0.05 compared to WT at the same time-points. (B) Effects of R+G+ on blood glucose. There was no difference in blood glucose levels between WT and IL-1-KO mice during R+G+ behavior. WT and IL-1-KO mice (n = 4–5) were subjected to R+G+, and blood was taken from the tail vein for blood glucose measurement. (C) Glycogen stores in IL-1-KO-MMs were strongly depleted during R+G+. WT and IL-1-KO mice (n = 12–16) were subjected to 1 h of R+G+ behavior or of R+G-, and MM glycogen content was measured. MMs from R-G- (i.e., resting, unrestrained) mice were also analyzed as a control. (D) Glucose uptake by IL-1-KO MMs (Left) was less than by WT MMs during R+G+, but not by the quadriceps femoris muscles (QFM, Right). WT and IL-1-KO mice (n = 10–12) were intraperitoneally injected with ^14^C-2DG after 1 h of R+G+ activity, and 30 min later skeletal muscles were removed and glucose uptake was measured. Experimental values are given as the mean ± SE. *P < 0.05, **P < 0.01.

In order to understand the function of IL-1α and β in MMs, we used IL-1α and IL-1β-double-deficient mice (IL-1 KO), and compared their indices of MM activity to those in WT mice. MM activity was measured by the reduction of strip weight due to gnawing (see *Materials and Methods*) under the R+G+ condition, and typical examples of gnawed plastic plates used for evaluating the MM activity are shown (Fig 4A, *right*). The MM activity of WT mice declined steadily with time (Fig 4A, *closed circles*). In contrast, the MM activity of IL-1-KO mice fell rapidly between 1 and 2 h after the start of R+G+ behavior, and then remained at a low level thereafter (Fig 4A, *open circles*). These results suggest that MMs from IL-1-KO mice are easily fatigued by R+G+ activity. However, it should be noted that there was no significant difference in the MM activity between WT and IL-1-KO mice during the initial 1 h of masticatory behavior.

### Blood Glucose Levels Are Not Involved in the Fatigability of IL-1-KO-MMs

To explore the mechanism behind the fatigability of MMs observed in IL-1-KO mice, we compared the blood glucose levels between WT and IL-1-KO mice during R+G+ behavior. Despite the marked difference in MM activity (Fig 4A), there was no significant difference in blood glucose levels between the two groups during 3 h of R+G+ (Fig 4B). These results suggest that the systemic glucose homeostasis remained intact, and blood glucose levels may not be involved in the fatigability of MMs in IL-1-KO mice.

### Derangement in Glucose Metabolism in MMs of IL-1-KO Mice During Masticatory Behavior

As described above, a significant reduction in MM activity was observed shortly after 1 h of R+G+ behavior in the IL-1-KO mice. We therefore imposed 1 h of R+G+ behavior on WT and IL-1-KO mice, and then analyzed MM activity to identify potential fatigue-related factor(s) unique to the IL-1-KO mice. We first compared intramuscular glycogen content between the MMs of WT and IL-1-KO mice. As shown in Fig 4C, 1 h of R+G+ behavior reduced glycogen content in WT MMs compared to that in non-masticating WT control mice (*closed bar*), although it did not reach statistical significance. However, a much greater reduction in glycogen content was observed in the MMs of IL-1-KO mice after 1 h of masticatory behavior, suggesting that intramuscular glycogen stores in IL-1-KO mice are depleted more rapidly by masticatory behavior than in WT mice.

In order to provide insight into the mechanism(s) underlying the rapid depletion of intramuscular glycogen observed in IL-1-KO mice, we next investigated the uptake of glucose into the MMs of WT and IL-1-KO mice during 1 h of R+G+ behavior using the ^14^C-2DG uptake assay [34]. As shown in Fig 4D, masticatory behavior significantly stimulated 2DG uptake into the working MMs of both WT and IL-1-KO mice, which was not detected in the non-masticating control groups (R+G- and R-G-) or in the nonworking muscles, the quadriceps femoris muscles (QFM). More importantly, the MM activity-dependent 2DG uptake was significantly less in MMs of IL-1-KO mice than that in the MMs of WT mice.

### IL-1 is Required for the MM-Activity-Induced IL-6 Production in MMs

**Fig 5:**
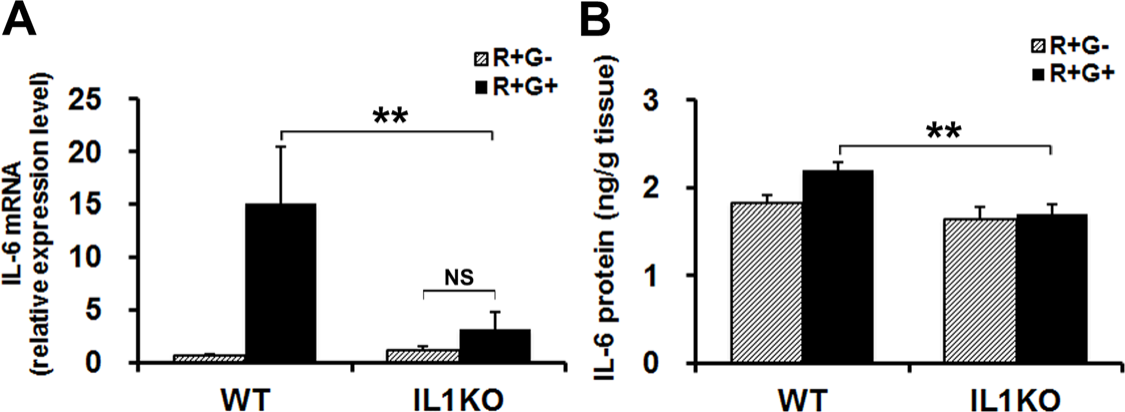
R+G+ induced IL-6 production via IL-1 stimulation in skeletal muscle. (A and B) R+G+ stimulates IL-6 production in WT MMs, but not in IL-1-KO MMs. WT and IL-1-KO mice were subjected to 1 h of R+G+ or of R+G-, and then IL-6 mRNA (A) and IL-6 protein (B) in their MMs were measured. Experimental values are given as the mean ± SE (n = 6 for A and n = 6–8 for B). **P < 0.01, NS: not significant.

Since IL-1 reportedly stimulates the production of IL-6, a major myokine affecting glucose metabolism in skeletal muscles [42,43], we next examined whether IL-6 is produced in the MMs of WT and IL-1-KO mice in response to R+G+. As shown in Fig 5A, 1 h of R+G+ behavior significantly increased IL-6 mRNA expression in the MMs of WT mice, but not in those of IL-1-KO mice. Consistent with these observations, the IL-6 protein level in MM homogenates from WT mice was significantly higher than in that of IL-1-KO mice after 1 h of masticatory behavior. However, a relatively high level of IL-6 under basal condition was observed, indicating that the MM activity-dependent increase in IL-6 observed in WT mice was low (Fig 5B).

### IL-1 Directly Stimulates IL-6 Production in C2C12 Myotubes

**Fig 6:**
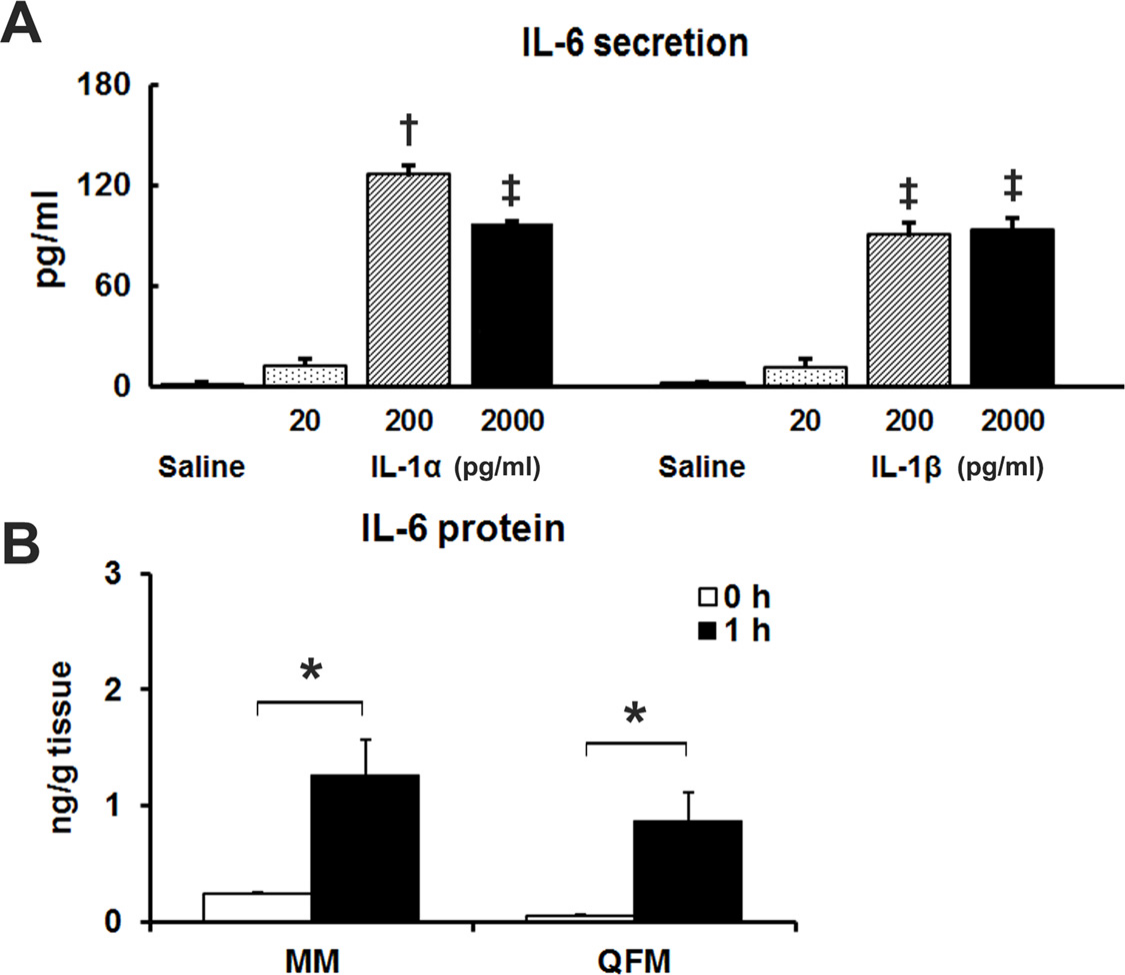
IL-1 stimulation induced IL-6 production. (A) C2C12 myotubes secrete IL-6 protein upon IL-1 administration. C2C12 myotubes were treated with various concentrations (pg/ml) of rm IL-1α and β for 2 h. IL-6 protein in the medium was subsequently measured via ELISA. Three wells of a 24-well plate were analyzed for each experimental treatment, and 2 separate experiments were performed. Experimental values are given as the mean ± SE (n = 6). ^a^ *P* <0.001 compared to all other groups, ^b^ *P* <0.001 compared to saline. (B) IL-1β injection induced IL-6 production in skeletal muscles *in vivo*. WT mice were intravenously injected with rm IL-1β at a dose of 5 ng/g BW, and IL-6 concentration was assayed at 0 and 1 h post-injection. In this experiment, QFM were also analyzed, confirming similar effects of IL-1β on skeletal muscles other than MMs. Experimental values are given as the mean ± SE (n = 5–6). *P < 0.05.

To further examine the potential involvement of IL-1 in the MM-activity-induced IL-6 production in skeletal muscle cells, we examined whether exogenous administration of IL-1α or β could stimulate IL-6 production in cultured C2C12 myotubes. As shown in Fig 6A, 200 pg/ml rm IL-1α and rm IL-1β induced increases in IL-6 secretion from C2C12 myotubes. While 200 pg/ml IL1α resulted in maximal IL-6 secretion in the present study, concentrations as low as 20 pg/ml rm IL-1α and β induced increases in IL-6 secretion, though not statistically significant.

### IL-1β Injection Induces IL-6 Production in Skeletal Muscles *In Vivo*

We next confirmed the induction of IL-6 by IL-1β in skeletal muscles *in vivo*. In total, 5 ng/g BW of rm IL-1β was intravenously injected. Thus, IL-1β may exert a similar effect on both MM and non-MM muscles, QFM (Fig 6B).

## Discussion

Key findings of this study are the low performance of MM activity during masticatory behavior in IL-1-KO mice, and concomitant rapid intramuscular glycogen depletion, perhaps resulting from compromised glucose uptake by working MMs. Furthermore, our data demonstrate that there is an increase in IL-1β, but not IL-1α mRNA, in working MMs in WT mice during masticatory behavior, which may be due to the locally recruited IL-1-producing cells in the perimysium of MMs. We also found that the MM-activity-induced IL-6 mRNA upregulation did not occur in IL-1-KO mice after 1 h of masticatory behavior, whereas IL-6 was induced in the MMs of WT mice upon mastication. In agreement with this, IL-6 mRNA expression was strongly induced by exogenous administration of IL-1 proteins to murine C2C12 myotubes, as well as in the MM and in the QFM by intravenous IL-1β injection in WT mice. Taken together, our data strongly suggest that IL-1β induces IL-6 secretion in working skeletal muscles including MMs, and stress the importance of localized IL-1-mediated interplay between autocrine and paracrine cytokines within active skeletal muscles for their performance and glucose homeostasis.

Normal exercise-induced increases in the plasma levels of IL-1α and/or IL-1β are reported to be very small, if any, in skeletal muscles [16–18], while strenuous exercise has been reported to induce IL-1β mRNA expression [19,44]. By utilizing our masticatory behavior (Restrained/Gnawing) model to examine the activity and/or fatigue in the MM [20,28,29], we observed that MM activity stimulates IL-1β mRNA expression, but increases in intramuscular IL-1β protein levels were below detection. However, we observed a localization of both IL-1α- and IL-1β-producing cells around the blood vessels in MMs after masticatory behavior, suggesting that IL-1 induced by MM activity is localized, or potentially limited to a fraction of the cells in MMs. Nevertheless, the low amounts of IL-1 induced appear to be functionally sufficient in supporting the MM activity upon proper glucose metabolism, which suggests that exogenous IL-1 administration would be inadequate to recover the partial abnormalities in glucose handling in IL1KO mice during masticatory behavior, a distinct local exercise. Our findings also suggest that IL-6 upregulation is evoked by locally increased IL-1 in response to muscle activity. Since IL-6 maintains muscle glucose homeostasis, the IL-1 induced in working MMs may indirectly support MM muscle activity through IL-6-maintained glucose homeostasis.

Interestingly, MMs from IL-1-KO appeared to be easily and rapidly fatigued by MM activity because of impaired glucose utilization by the working skeletal muscles. In previous *in vitro* studies, it has been reported that IL-1 failed to directly facilitate glucose uptake in murine muscle cells [45,46]. In contrast, *in vivo* studies including our own previous work, have demonstrated that exogenously injected IL-1 (either IL-1α or IL-1β) induced transient hypoglycemia [47–49] by modifying systemic levels of hormones such as insulin and glucagon [49]. Thus, it is generally considered that *in vivo*, IL-1 regulates glucose metabolism in skeletal muscle tissues through indirect pathways.

IL-6, a typical myokine, plays an important role in enhancing glucose utilization in skeletal muscles during exercise [16–18,50], and is generally induced by IL-1 [26,51]. We previously demonstrated that R+G+ stimulates IL-6 production in MMs, and that IL-6 helps to prevent muscle fatigue via the regulation of muscle glucose metabolism [20]. Here, we found that following masticatory behavior, the IL-6 protein level was significantly lower in the MMs of IL-1-KO mice than in those of WT mice. Indeed, low concentrations of both IL-1α and β directly stimulate IL-6 production in cultured skeletal muscle cells. These findings suggest that the masticatory dysfunction along with muscle fatigue observed in IL-1-KO mice herein may be caused by impairment of glucose utilization due to compromised IL-6 synthesis in the working muscles. Thus, our findings support the view that an exercise-induced local release of IL-6 from skeletal muscles may be partly dependent on IL-1 induction, and the released IL-6 may consequently support the muscle’s endurance against exercise-induced fatigue or exhaustion by contributing to the maintenance of glucose homeostasis.

Unlike the high expression of IL-6 in skeletal muscle cells, IL-1 expression in skeletal muscles seems to be very low. We indeed failed to detect increased IL-1 expression in C2C12 myotubes using qPCR and ELISA. However, we observed that the myotubes were capable of expressing adequate amounts of IL-6 when stimulated by low concentrations of IL-1 (either IL-1α or IL-1β), which is comparable to IL-6 induced by electrical muscle stimulation (data not shown) [52]. Interestingly, we found that IL-1 producing cells appeared around blood vessels in perimysium after MM activity. It is a well-known phenomenon that typical IL-1 producing cells such as neutrophils and monocytes are recruited into skeletal muscle tissues during the exercise [44]. Herein, Gr-1, a major marker for granulocytes, could be clearly distinguished in IL-1α/β-producing cells; IL-1β-positive cells co-expressing Gr-1 signals were concluded to be migrating neutrophils [38]. However, IL-1α-positive cells acted as other immune cells like mast cells, but not neutrophils, owing to their absence of Gr-1 signals [38,53]. Considering the above observations, it would be of great interest to further identify the phenotype(s) of the IL-1-positive cells and the mechanism underlying their recruitment, as well as the precise role of the IL-1-positive cells in muscle physiology in future studies.

**Fig 7:**
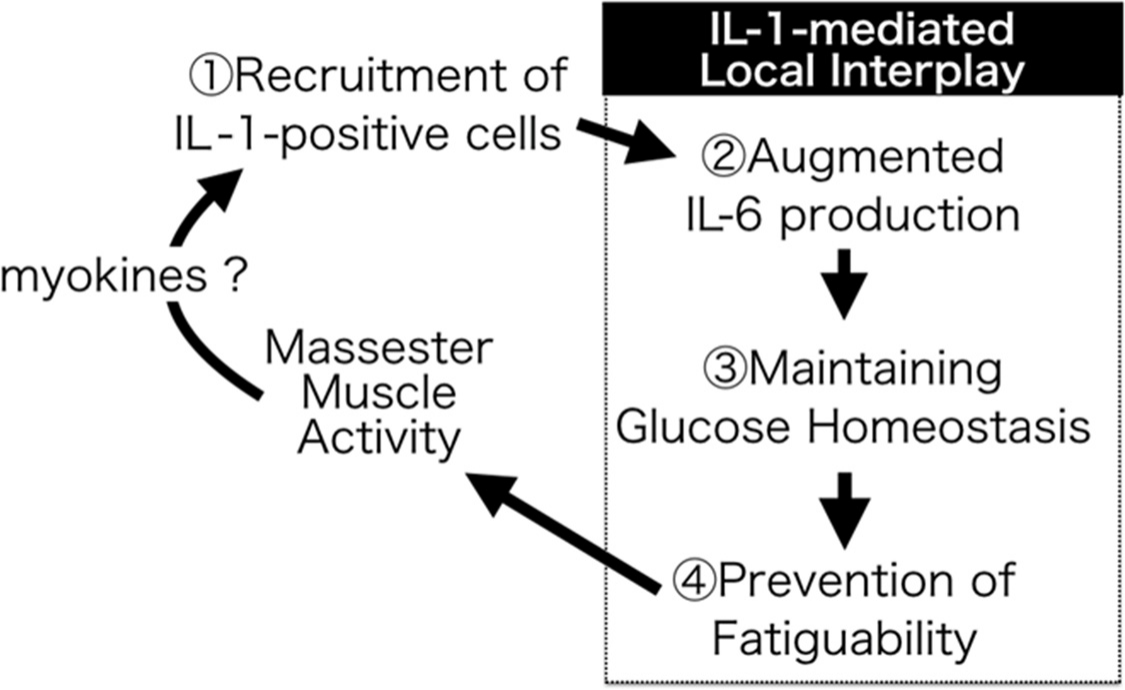
Summary of the roles of IL-1 in MM activity. Masseter muscle (MM) activity locally results in both IL-1α/β induction owing to the recruitment of IL-1-producing cells, which might be caused by myokines and/or mechano-stimulations. IL-1 released from migrating cells protects MMs against fatigue via the augmented production of IL-6, a major mediator of muscle glucose metabolism during its contractile activity.

The present findings placed in the context of prior research suggest that IL-1 produced during mastication may support continuing MM activity via the production of IL-6, a major mediator of muscle glucose metabolism during physical activity (Fig 7). Chronic muscle fatigue seen in various musculoskeletal disorders, is strongly associated with poor mobility, functional limitations, and mortality [13,14]. Similarly, MM fatigue observed in TMD patients directly affects their quality of life due to chewing difficulties [9,10]. Dysregulation of myokines such as IL-6 and IL-1 may provide crucial therapeutic targets to improve pathological muscle fatigue.

## Supporting Information

S1 Fig PCR strategies and primers for IL-1-KO mice genotyping. (A) Schematic structures of the wild-type (WT) locus and mutant (MT) locus on the IL1A and IL1B genes are shown. Numbered solid boxes depict exons of IL1A or IL1B. LacZ-pA cassettes inserted into IL-1 genes are indicated by the gray boxes. Primers and their sequences were as follows: P1: 5′-CTG CCA GGG CTC CAT CAT GAG AC-3′; P2: 5′-GAG GTG CTG TTT CTG GTC TTC ACC-3′; P3: 5′-CAC ATA TCC AGC ACT CTG CTT TCA G-3′; P4: 5′-GGT CAGT GTG TGG GTT GCC TTA TC-3′. (B) The upper band (720 bp) indicates the existence of wild-type allele of IL1B. The middle band including the MT site of IL1B genes (420 bp) and the lower band for the MT of IL1A (560 bp) were detected only in IL-1-KO mice. Then, the wild-type allele of IL1A (1,540 bp) was confirmed by an additional RT-PCR to distinguish the heterozygous mice.
(TIF)
