# Supplementary figures and images for: Involvement of IL-1 in the Maintenance of Masseter Muscle Activity and Glucose Homeostasis
Source: PLoS One. 2015 Nov 24;10(11):e0143635. doi: 10.1371/journal.pone.0143635 (PMC4658060; doi:10.1371/journal.pone.0143635)

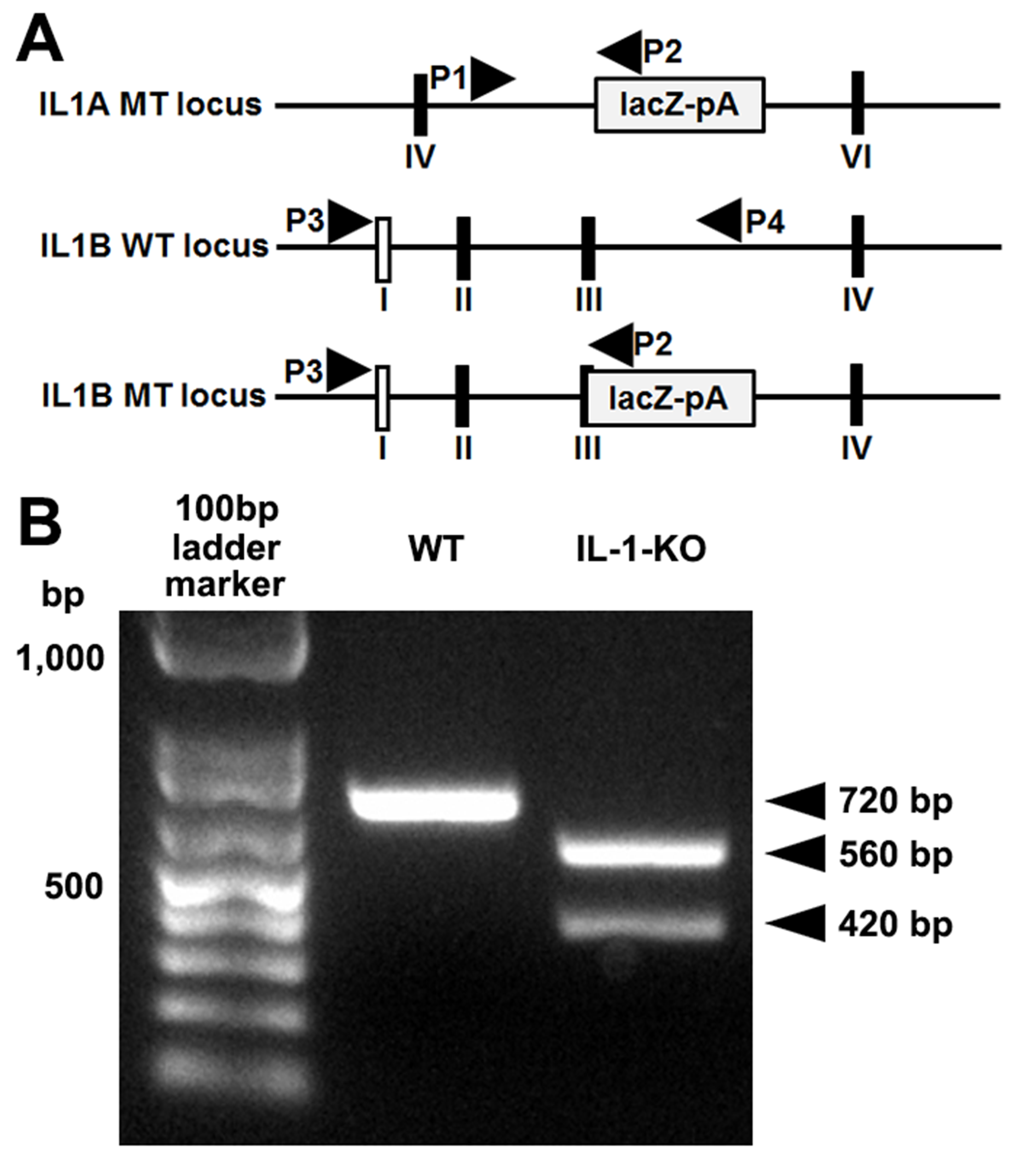

Supplement: S1 Fig — (A) Schematic structures of the wild-type (WT) locus and mutant (MT) locus on the IL1A and IL1B genes are shown. Numbered solid boxes depict exons of IL1A or IL1B. LacZ-pA cassettes inserted into IL-1 genes are indicated by the gray boxes. Primers and their sequences were as follows: P1: 5′-CTG CCA GGG CTC CAT CAT GAG AC-3′; P2: 5′-GAG GTG CTG TTT CTG GTC TTC ACC-3′; P3: 5′-CAC ATA TCC AGC ACT CTG CTT TCA G-3′; P4: 5′-GGT CAGT GTG TGG GTT GCC TTA TC-3′. (B) The upper band (720 bp) indicates the existence of wild-type allele of IL1B. The middle band including the MT site of IL1B genes (420 bp) and the lower band for the MT of IL1A (560 bp) were detected only in IL-1-KO mice. Then, the wild-type allele of IL1A (1,540 bp) was confirmed by an additional RT-PCR to distinguish the heterozygous mice. (TIF) [file pone.0143635.s001.tif]
